# Development and validation of a RNA binding protein-associated prognostic model for head and neck squamous cell carcinoma

**DOI:** 10.18632/aging.202848

**Published:** 2021-03-24

**Authors:** Xiuping Yang, Baoai Han, Runshi Zhang, Yuan Su, Davood K. Hosseini, Han Wu, Minlan Yang, Haiying Sun

**Affiliations:** 1 Department of Otorhinolaryngology, Union Hospital, Tongji Medical College, Huazhong University of Science and Technology, Wuhan 430022, China; 2 Department of Otorhinolaryngology, Head and Neck Surgery, Zhongnan Hospital of Wuhan University, Wuhan 430071, China; 3 Department of Clinical Laboratory, Xi'an No. 1 Hospital, Xi'an 710000, China; 4 Department of Clinical Laboratory, Xi'an Labor Union Hospital, Xi'an 710000, China; 5 Department of Internal Medicine, Hackensack University Medical Center, Hackensack, NJ 07601, USA

**Keywords:** HNSCC, TCGA database, prognostic model, RBPs, OS

## Abstract

Evidence shows that defects in RNA-binding proteins (RBPs) are closely related to the occurrence and development of HNSCC. We obtained 502 tumors and 44 normal samples from the TCGA database, among which 190 differentially expressed RBPs were screened. Finally, a prognostic model containing nine RBPs (*CELF2, CPEB1, DDX39B, EIF3L, EZH2, KHDRBS3, RNASE10, RNASE3* and *SIDT1*) was produced. Further analysis showed that the overall survival rate in the high-risk group was lower than that in the low-risk group. The area under the ROC curve (AUC) in the training and testing groups was significant (3-year AUC, 0.735 vs 0.796; 5-year AUC, 0.821 vs 0.804). In addition, a comprehensive analysis of nine identified RBPs showed that most of them were related to the OS of HNSCC patients, and three of them (*CELF2, EZH2,* and *SIDT1*) were differentially expressed in HNSCC and control tissues at the protein level. In addition, our data revealed that the identified RBPs are highly interconnected, with high frequency copy number changes in HNSCC samples. GSEA indicated that the abnormal biological processes related to RNA and the activation of some classical tumor signaling pathways were important driving forces for the development of HNSCC. Our results provide novel insights into the pathogenesis of HNSCC, among which nine RBP markers have potential application value in clinical decision-making and individualized treatment of HNSCC.

## INTRODUCTION

Head and neck malignancies are among the most common diseases known to affect millions of people worldwide with high mortality rates. The most common type of head and neck cancer is squamous cell carcinoma, which accounts for approximately 95% of all head and neck cancers [1, 2]. Despite great advances in diagnostic and treatment methods over the past few years, the average five-year survival rate for head and neck squamous cell carcinoma (HNSCC) has not changed significantly, remaining at 50% [3]. Most patients are already in the advanced stage when they are diagnosed, which may be the reason for the high mortality rate in HNSCC patients. The diagnosis of HNSCC mainly relies on histopathological analysis and imaging evaluation, which makes it difficult to achieve early detection [4]. Thus, it is requisite to explore novel diagnostic methods for the intervention and discovery of HNSCC.

RNA-binding proteins (RBPs) are important members of posttranscriptional regulation. Recent studies revealed the regulatory role of RBPs in cell differentiation, proliferation, apoptosis, metabolism and other biological processes related to both the development and progression of cancer [5–7]. To date, 1542 RBPs have been experimentally confirmed to exist in the human genome, accounting for 7.5% of protein-coding genes. RBPs involve almost all steps of the posttranscriptional regulatory layer. RBPs can regulate posttranscriptional mRNA stability, RNA processing, splicing, localization, translocation, monitoring, decay and translation by binding to target RNA [8]. Therefore, changes in RBP expression can affect many aspects of RNA metabolism. RBPs also play a significant role in the occurrence and development of cancer. Cancer is a complex and heterogeneous disease. Tumor cells can regulate protein expression levels by hijacking posttranscriptional regulation to make them better adapt to the microenvironment [9, 10]. It was found that RBPs were dysfunctional in different types of cancer, which affected the expression and function of tumor suppressor proteins and oncoproteins. For example, tristetraprolin (*TTP*) is an RNA-binding protein encoded by the zinc finger protein 36 *(ZFP36*) gene. Suswam et al. found that *TTP* was overexpressed in malignant glioma cells, significantly promoting the degradation of VEGF and IL-8 mRNA and inhibiting the growth and invasion of tumor cells [11]. Gebeshube et al found that miR-29A inhibited the expression of *TTP* and cooperated with Ras signaling to promote the development of breast cancer. Overexpression of *TTP* in human breast cancer cells can significantly inhibit the invasion, metastasis, and proliferation of breast cancer cells [12]. *QKI-5* is a member of the RNA-binding protein family. Studies have found that *QKI-5* mainly reduces MAPK/ERK signaling pathways and reduces the expression of p-ERK, thereby inhibiting the proliferation of kidney cancer cells [13]. Studies have discovered that RNA-binding protein 24 (RBM24) is oftentimes downregulated in nasopharyngeal carcinoma (NPC). The restoration of RBM24 expression inhibited the migration, invasion and proliferation of NPC cells and hindered the transfer and colonization of mice [14]. Therefore, deciphering the intricate network of interactions between RBPs and their cancer-related RNA targets will offer a better understanding of tumor biology and may disclose novel cancer treatment targets. At present, research on RBPs and HNSCC is limited, and only a small portion of RBPs have been studied in depth and detected to play a vital role in head and neck cancer.

In view of this situation, we tried to systematically analyze the potential value of RBPs in HNSCC by integrating a full set of RBPs and clinical information obtained from the TCGA database. Firstly we identified the differentially expressed RBPs (DERBPs) in HNSCC and constructed a risk prediction model of DERBPs. Then, Least absolute shrinkage and selection operator (LASSO) regression and Cox regression analyses were used to optimize the model, and DERBPs related to the OS rate was selected. We used these DERBPs to establish a Cox regression model and used ROC curve analysis to evaluate the sensitivity and specificity of the model. According to our data, this specific model can predict patients' prognosis accurately. These findings not only provide new insights into the pathogenesis of HNSCC, but also provide an effective biomarker-based multi-dimensional strategy for HNSCC patients' prognosis prediction.

## RESULTS

### Flow chart of our study

**Figure 1:**
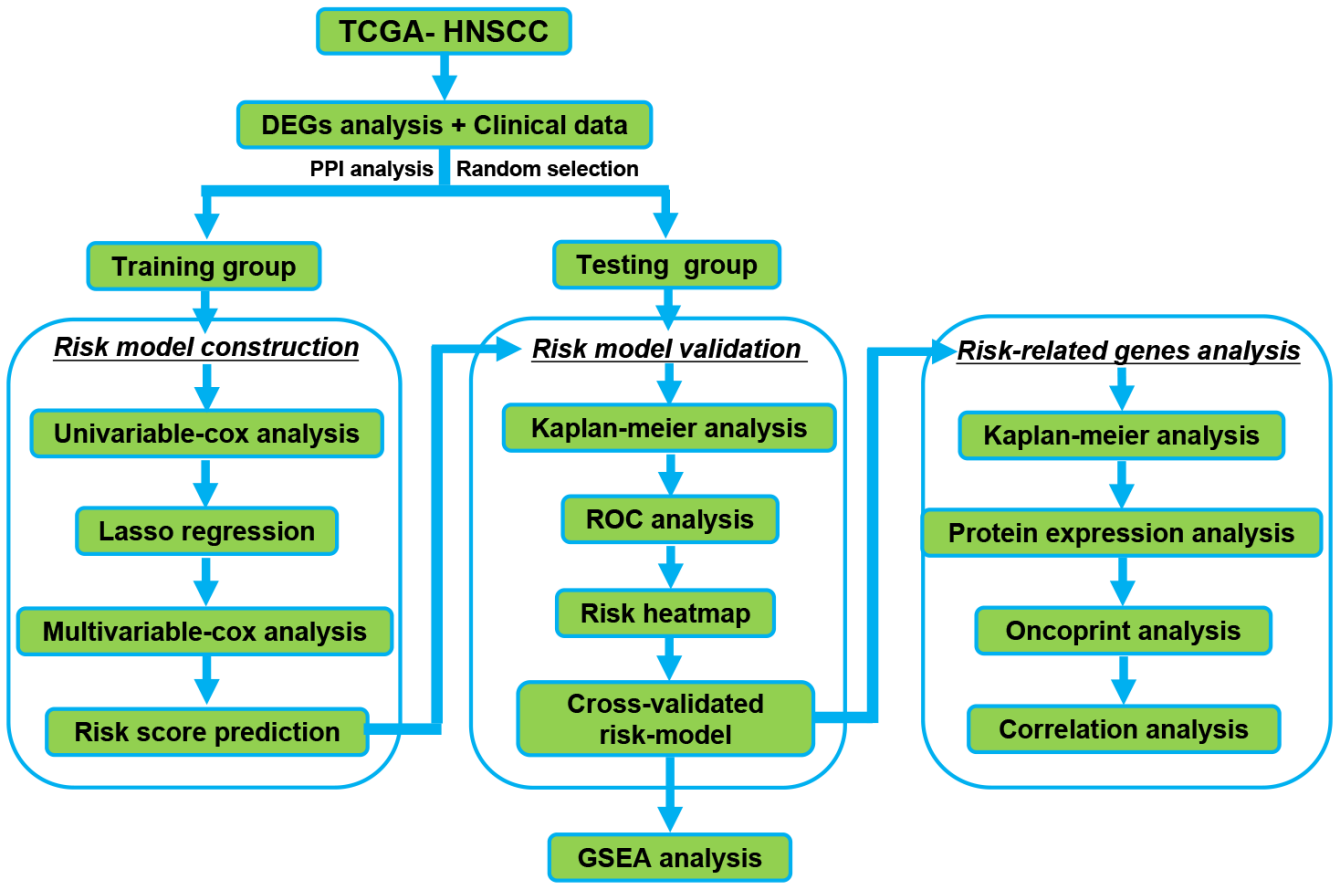
**The Flowchart for identification the survival-related RBPs in HNSCC.** RBPs: RNA binding proteins. HNSCC: Head and Neck Squamous Cell Carcinoma.

Figure 1 shows the detailed workflow of the study. Firstly, DERBPs that are differentially expressed between normal samples and HNSCC are found. Then, we used the training group to construct a specific prognostic model. The prognostic model was further confirmed and optimized in the testing group. The prediction power of these models was checked using time-dependent ROC analysis. Then, the RBPs in the prognosis model were comprehensively verified. The prognostic value and key role of RBPs was analyzed by our study systematically in HNSCC.

### Differential expression and functional annotation analysis of RBPs in HNSCC

**Figure 2:**
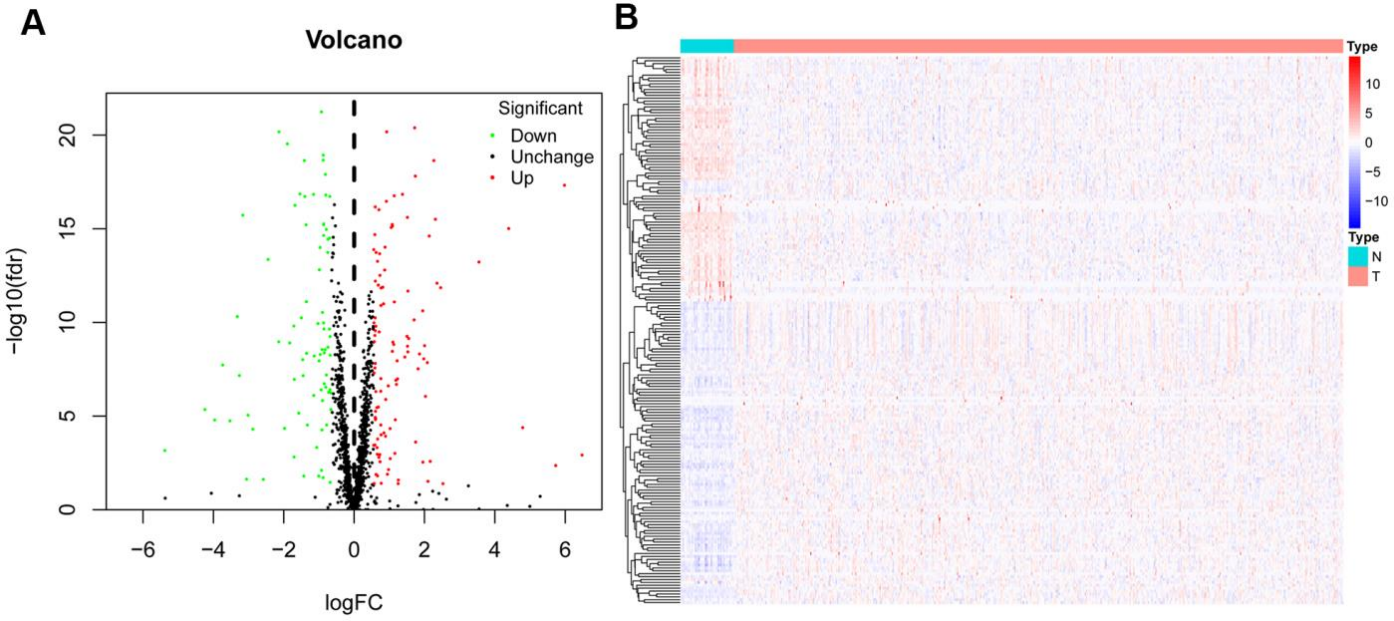
**Differentially expressed RBPs in HNSCC and non-tumour samples.** The volcano plot (**A**) and Clustered heatmap (**B**) of differentially expressed RBPs in HNSCC and normal tissues.

**Figure 3:**
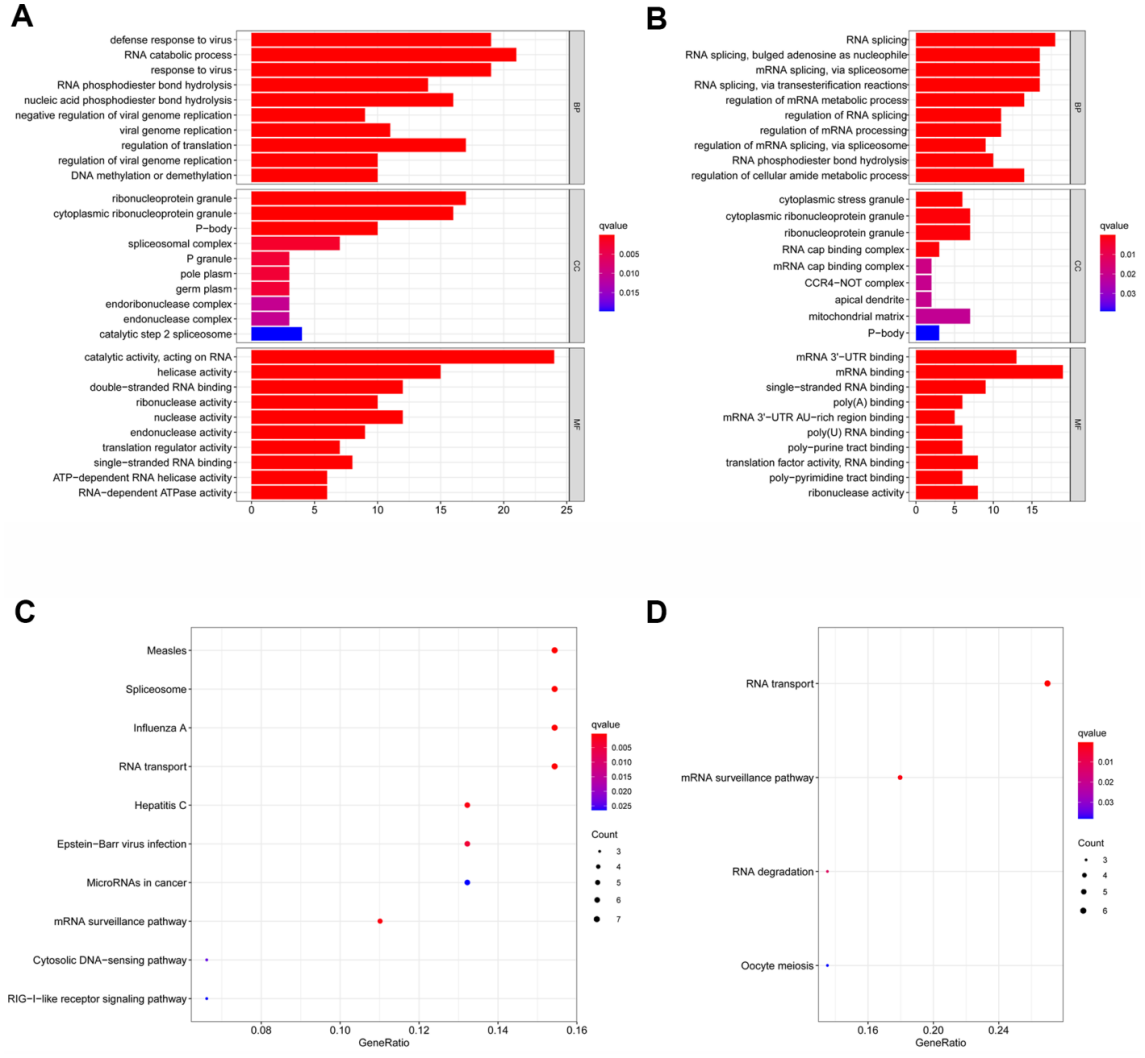
**Gene ontology (GO) and kyoto encyclopedia of genes and genomes (KEGG) analysis of the differentially expressed RBPs.** Results of GO functional annotation analysis of up (**A**) and down (**B**) regulated differentially expressed RBPs; Results of KEGG pathways enrichment analyses of up (**C**) and down (**D**) regulated differentially expressed RBPs. The dot size represents the enriched gene number.

The corresponding clinical data and mRNA expression data of 44 nontumor samples and 502 HNSCC tissue samples were downloaded from TCGA database (Table 1). After abstracting 1495 RBP expression values, we acquired differentially expressed RBPs and showed the expression pattern of differentially expressed RBPs in HNSCC and non-tumor tissues by volcano map and thermogram. In HNSCC, 190 differentially expressed genes were obtained in tumour tissues, of which 109 genes were upregulated and 81 genes were downregulated (Figure 2). Then, to understand the biological characteristics of these genes, we performed functional enrichment analysis on upregulated and downregulated RBPs. Figure 3 summarizes the GO terms and enriched KEGG pathways of these genes. In HNSCC, our results indicate that in the related biological processes, upregulated RBPs are significantly enriched in nucleic acid phosphodiester bond hydrolysis, RNA catabolic process, negative regulation of viral genome replication, regulation of translation and DNA methylation or demethylation (Figure 3A), while downregulated RBPs are significantly enriched in the regulation of RNA splicing, regulation of the mRNA metabolic process, regulation of mRNA splicing via the spliceosome, regulation of mRNA processing, and regulation of cellular amide metabolic process (Figure 3B). In terms of molecular function, upregulated RBPs were significantly enriched in acting on RNA, catalytic activity, double-stranded RNA binding, helicase activity, ribonuclease activity and nuclease activity (Figure 3A), while downregulated RBPs were significantly enriched in mRNA binding, mRNA 3' UTR binding, poly(A) binding, single-stranded RNA binding and mRNA 3' UTR AU-rich region binding (Figure 3B). Through cell composition (CC) analysis, we found that the upregulated RBPs were mainly enriched in ribonucleoprotein granules, cytoplasmic ribonucleoprotein granules, P-bodies, spliceosomal complexes and P granules (Figure 3A), while the downregulated RBPs were significantly enriched in cytoplasmic stress granules, cytoplasmic ribonucleoprotein granules, ribonucleoprotein granules, RNA cap binding complexes and mRNA cap binding complexes (Figure 3B). In addition, the results of KEGG pathway enrichment analysis of differentially expressed RBPs showed that the upregulated RBPs were enriched in the spliceosome, RNA transport, the mRNA surveillance pathway, the RIG-I-like receptor signaling pathway and microRNAs in cancer (Figure 3C), while the downregulated RBPs were enriched in RNA transport, the mRNA surveillance pathway, RNA degradation and progesterone-mediated oocyte maturation (Figure 3D).

**Table 1:** Clinicopathological parameters of HNSCC patients in the TCGA database.

**Clinical parameters**	**Variable**	**Total (528)**	**Percentages (%)**
**Age**	<=65	345	65.3%
	>65	182	34.5%
	Unknow	1	0.2%
**Gender**	Female	142	26.9%
	Male	386	73.1%
**Grade**	G1	63	11.9%
	G2	311	58.9%
	G3	125	23.7%
	G4	7	1.3%
	GX	22	4.2%
**Pathological stage**	Stage ^I^	27	5.1%
	Stage ^II^	74	14.0%
	Stage ^III^	82	15.5%
	Stage ^IV^	270	51.1%
	Unknow	75	14.2%
**T stage**	T0	1	0.2%
	T1	49	9.3%
	T2	140	26.5%
	T3	101	19.1%
	T4	175	33.1%
	TX	62	11.7%
**M stage**	M0	191	36.2%
	M1	1	0.2%
	MX	336	63.6%
**N stage**	N0	180	34.1%
	N1	68	12.9%
	N2	172	32.6%
	N3	8	1.5%
	NX	100	18.9%
**Survival status**	Dead	199	37.7%
	Alive	329	62.3%

Abbreviations: T, Tumor; M, Metastasis; N, Node.

### Construction of the PPI network and screening of key modules

**Figure 4:**
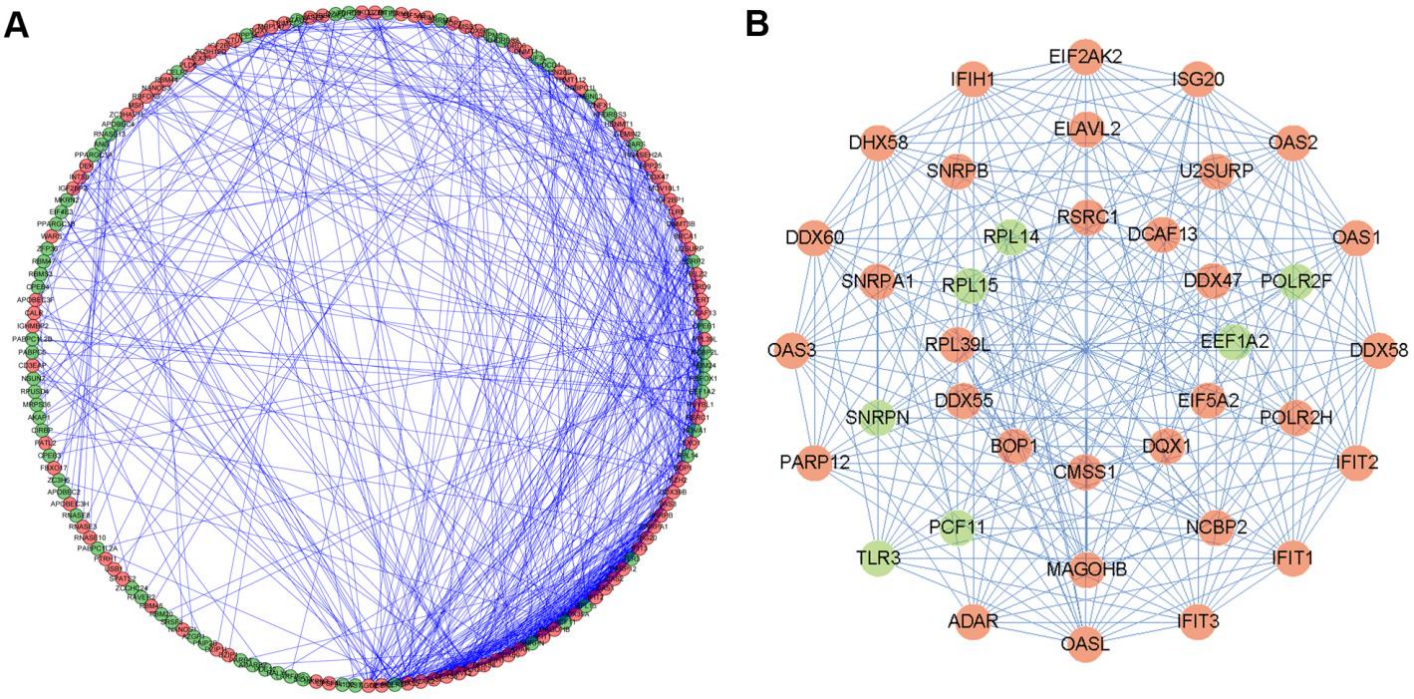
**Protein-protein interaction (PPI) network and modules analysis.** (**A**) PPI network of differentially expressed RBPs; (**B**) 3 critical modules from PPI network. The outer circle is critical module 1, the middle circle is critical module 2, and the inner circle is critical module 3. Red circles: up regulation RBPs; Green circles: down regulation RBPs.

To better understand the potential molecular functions of these differentially expressed RBPs in HNSCC, a PPI network was constructed using the Cytoscape software and STRING database. The PPI network consists of 161 nodes and 581 edges (Figure 4A). Then, we further analyzed the co-expression network and used the plug-in pattern in Cytoscape to detect the potential key modules and determine the first three important modules (Figure 4B). Module 1 consists of 16 nodes and 113 edges, module 2 consists of 10 nodes and 45 edges, and module 3 consists of 12 nodes and 35 edges. Functional enrichment showed that the genes of module 1 were mainly enriched in the regulation of multi-organism processes, RNA binding and nucleic acid binding. The genes of module 2 were significantly enriched in mRNA splicing, the spliceosome, and the mRNA surveillance pathway. However, the genes of module 3 were significantly enriched in rRNA processing, RNA processing, and translation (Supplementary Table 1).

### Construction and verification of the HNSCC-specific predictive prognosis model

**Figure 5:**
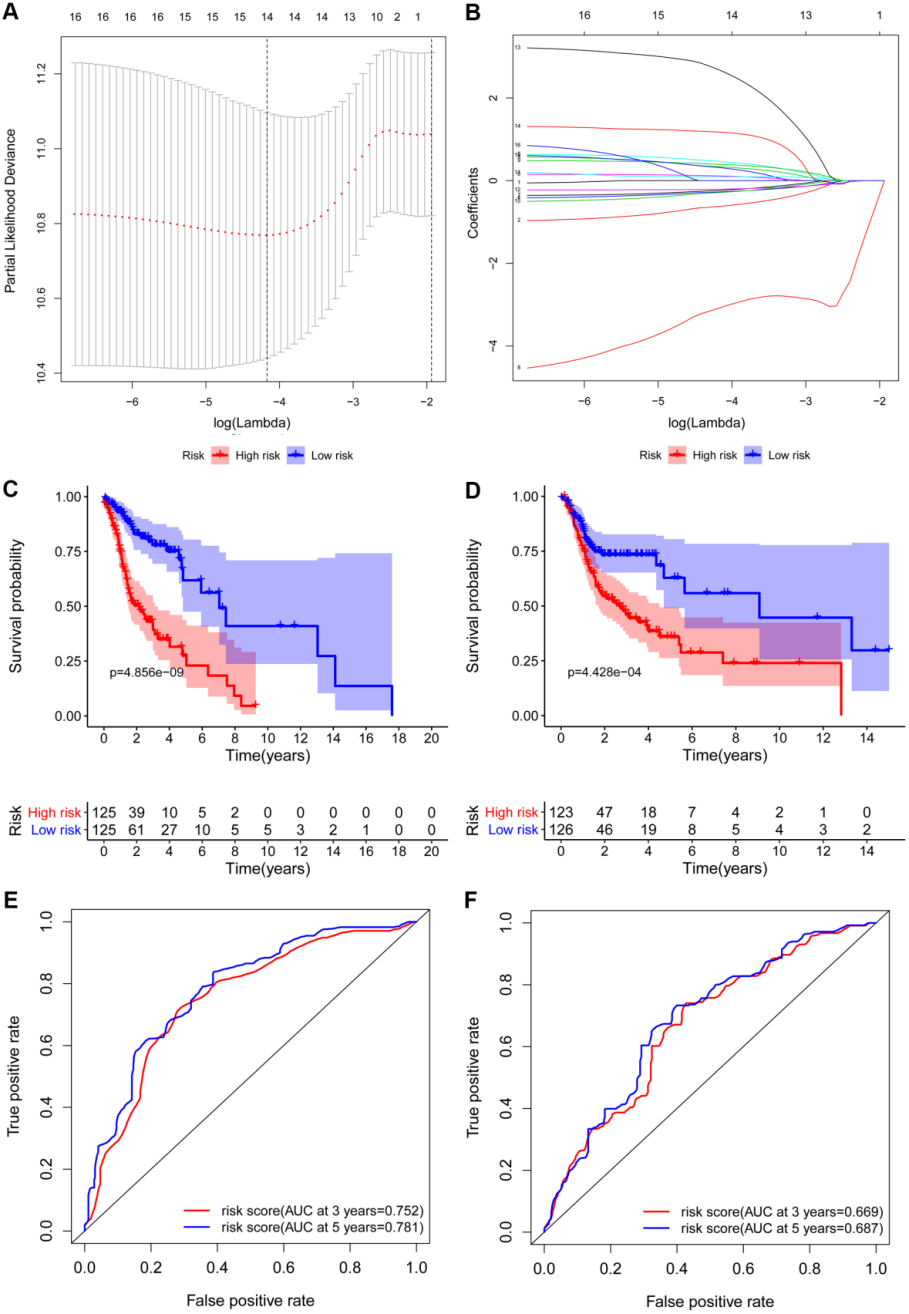
**Construction and validation of the prognostic risk model in HNSCC patients.** (**A**) Screening of optimal parameter (lambda) at which the dotted vertical lines were drawn. (**B**) Lasso coefficient profiles of the candidate RBPs with non-zero coefficients determined by the optimal lambda. Kaplan-Meier plot of the high-risk (red) and low-risk (blue) HNSCC patients in the training group (**C**) and testing group (**D**). The 3-year (red) and 5-year (blue) ROC curves in the training group (**E**) and testing group (**F**) of HNSCC patients.

**Figure 6:**
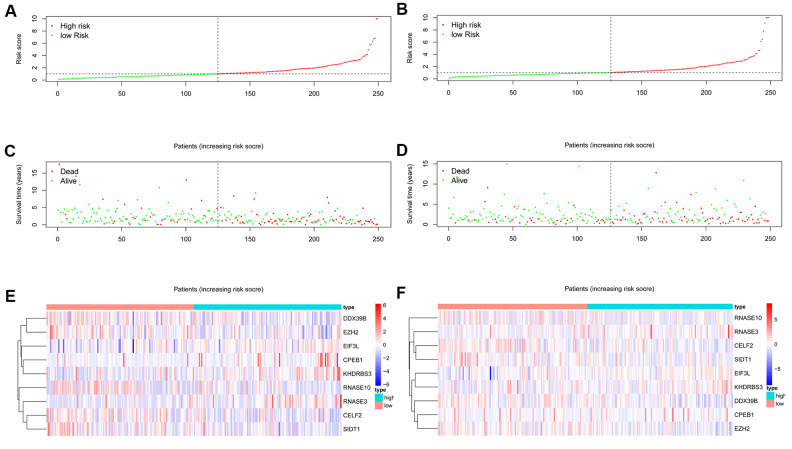
**RBPs-related prognostic characteristics in patients with HNSCC.** Risk score distribution of HNSCC patients with different risks in the training group (**A**) and testing group (**B**) (low, green; high, red). Dot plots showing the survival time and risk score in training group (**C**) and testing group (**D**). The heatmap of the 9 key genes expression profiles in the training group (**E**) and testing group (**F**) (low, blue; high, red).

A total of 161 key RBPs with differential expression were identified from the PPI network. In order to study the relationship between the expression of RBPs and the prognosis of HNSCC patients, we established the prognosis model of HNSCC patients in the training group. Univariate Cox regression analysis was initially executed to acquire genes that were significantly related to prognosis, and LASSO regression and multivariate Cox regression analyses were then used to create the final prognosis model (Table 2, and Figure 5A, 5B). After creating the prognosis model, the patients were divided into a low-risk group and a high-risk group, and Kaplan-Meier survival analysis was then carried out on the testing set and training set. The results demonstrated that the overall survival time of patients with high risk score was obviously poorer than those with low risk score in the HNSCC dataset (Figure 5C, 5D). In HNSCC patients, a 9-gene model (*CELF2, CPEB1, DDX39B, EIF3L, EZH2, KHDRBS3, RNASE10, RNASE3* and *SIDT1*) was successfully obtained. Using this model to anticipate the risk score of each patient, we found that *CPEB1, EIF3L, KHDRBS3* and *RNASE3* are positive risk-related genes, whereas *CELF2, DDX39B, EZH2, RNASE10* and *SIDT1* are negative risk-related genes. The AUC (area under the ROC curve) in the training and testing groups was remarkable (3-year AUC, 0.752 vs. 0.669; 5-year AUC, 0.781 vs. 0.687) (Figure 5E, 5F). The model can accurately predict the OS of HNSCC patients. Besides, we ranked all HNSCC patients according to risk score to analyze the survival rate distribution. From the scatter plot, we can see the survival status of patients with different risk score; the mortality rate of patients rises with increasing risk score. Heat maps show that the expression of RBPs is related to the increase of patient risk score (Figure 6A–6F).

**Table 2:** The nine selected RNA binding proteins.

**Id**	**Coef**	**HR**	**HR.95L**	**HR.95H**	**P value**
CELF2	-0.8124	0.4438	0.2999	0.6567	0.0000
CPEB1	0.5103	1.6658	0.863	3.2156	0.1283
DDX39B	-0.3808	0.6833	0.4723	0.9885	0.0432
EIF3L	0.6228	1.8642	1.3168	2.6391	0.0004
EZH2	-0.4283	0.6516	0.4477	0.9483	0.0253
KHDRBS3	0.4804	1.6167	1.1194	2.3351	0.0104
RNASE10	-0.2886	0.7493	0.6131	0.9159	0.0048
RNASE3	3.3622	28.8515	3.3959	245.1251	0.0021
SIDT1	-0.3783	0.685	0.4009	1.1705	0.1664

Abbreviations: HR, hazard ratio; HR.95 L/H, 95 % confidence interval of the hazard ratio.

### The prognosis model of HNSCC patients is independently associated with OS

**Figure 7:**
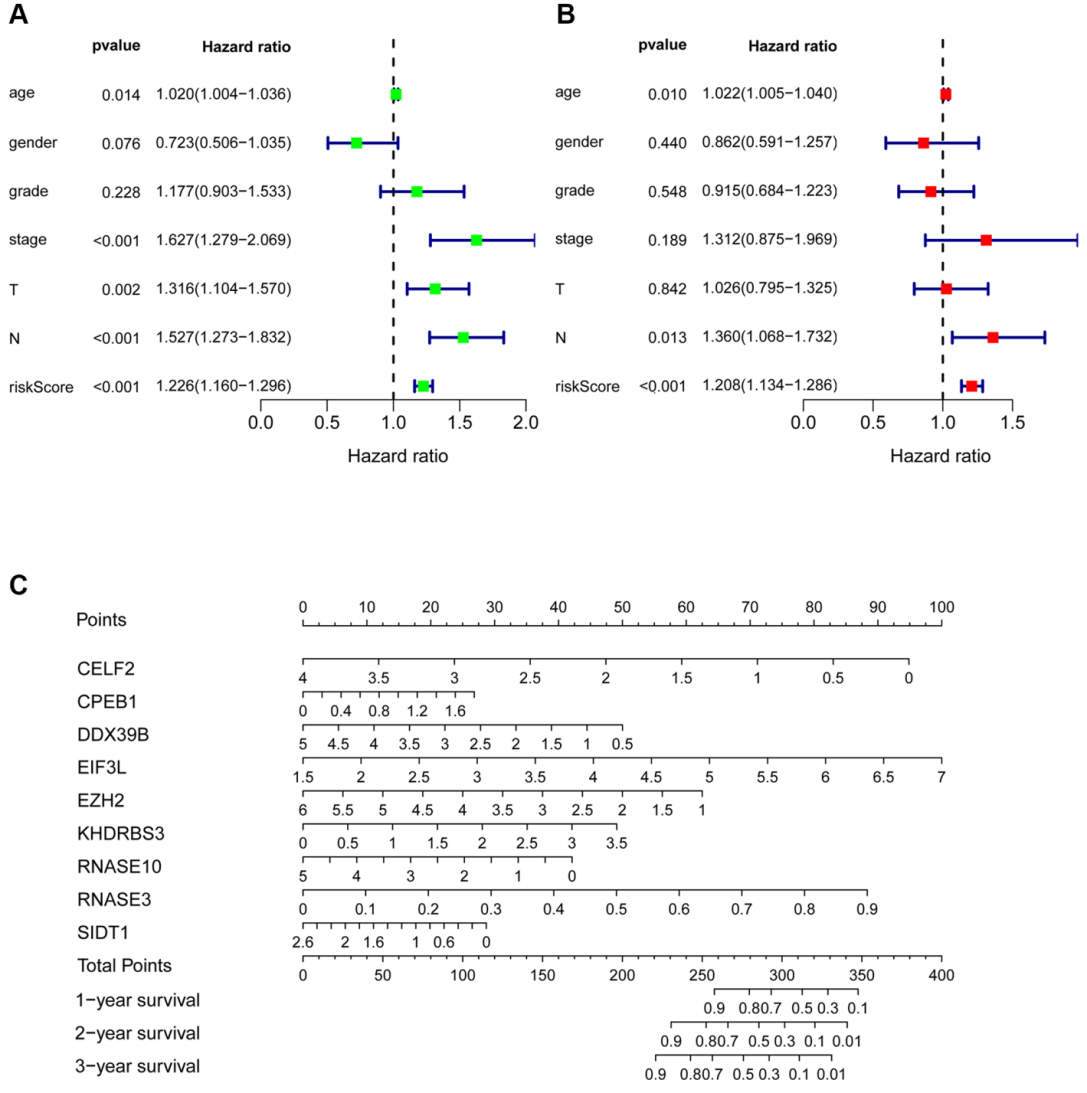
**The prognostic value of different clinical parameters.** Univariate (**A**) and multivariate (**B**) Cox regression analyses of OS in HNSCC. (**C**) Nomogram for predicting 1-, 3-, and 5-year OS of HNSCC patients.

Cox regression analysis was used to analyze the correlation between OS and clinical parameters such as histological grade, age, risk score and pathological stage. Univariate Cox regression analysis demonstrated that the histological grade, age, pathological stage, and risk score of patients were associated with OS (P <0.05). However, by multivariate regression analysis, we found that only risk score, N stage and age were independent prognostic factors associated with OS (P <0.05) (Figure 7A, 7B). At the same time, to construct a quantitative model of the prognosis of HNSCC patients, we also combined 9 RBP markers to construct a nomogram (Figure 7C and Supplementary Figure 3). Based on multivariate Cox analysis, the point scale of the nomogram was used to assign points to each variable. We drew a horizontal line to determine the points of each variable, calculated the total points of each patient by summing the points of all variables, and then standardized the total points of each patient to a distribution of 0 to 100. By constructing a vertical line between each prognostic axis and total point axis, the estimated 1-, 3-, and 5-year survival rates of HNSCC patients could be calculated, which may help relevant practitioners make clinical decisions for HNSCC patients. Our consequences suggest that the established specific prognostic models and genes can be used to predict the OS of HNSCC patients.

### Comprehensive analysis of genes in the RBP prognosis model

**Figure 8:**
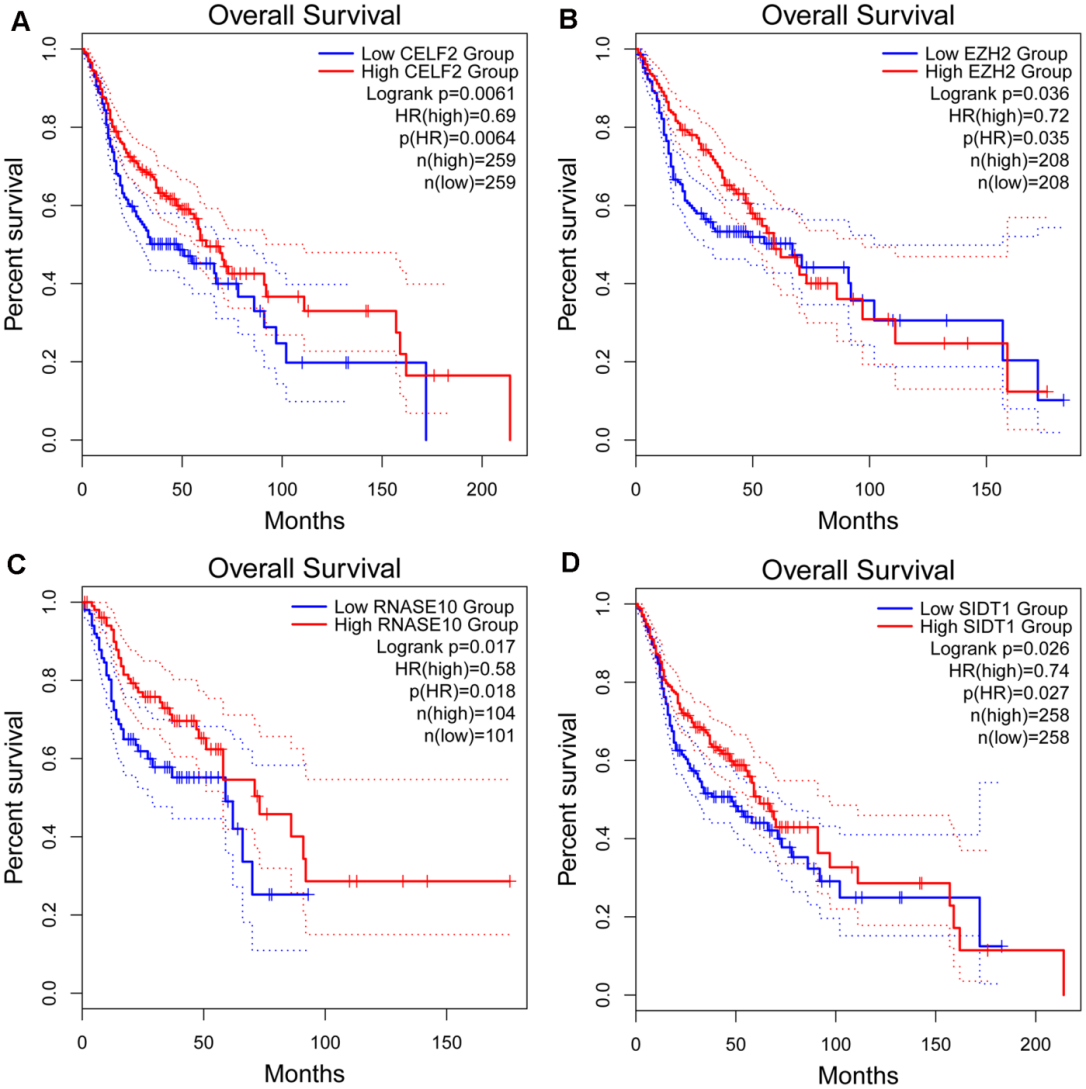
**Kaplan-Meier analyses of ARGs in prognostic model.** Kaplan-Meier analyses of (**A**) CELF2, (**B**) EZH2, (**C**) RNASE10 and (**D**) SIDT1. The statistical significance was determined by *Log-rank* test.

**Figure 9:**
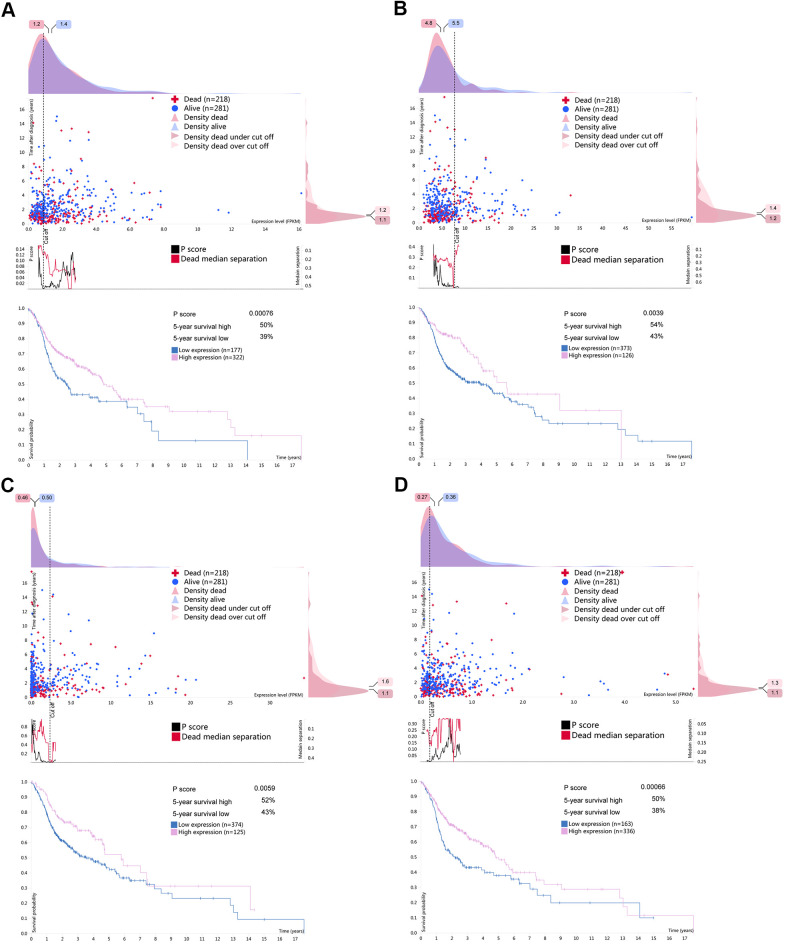
**Survival associated RBPs expression in the human protein atlas database (HPA).** Kaplan-Meier curves of survival associated (**A**) CELF2, (**B**) EZH2, (**C**) RNASE10 and (**D**) SIDT1 for HNSCC patients. Pink line indicates high expression group while blue line indicates low expression group.

**Figure 10:**
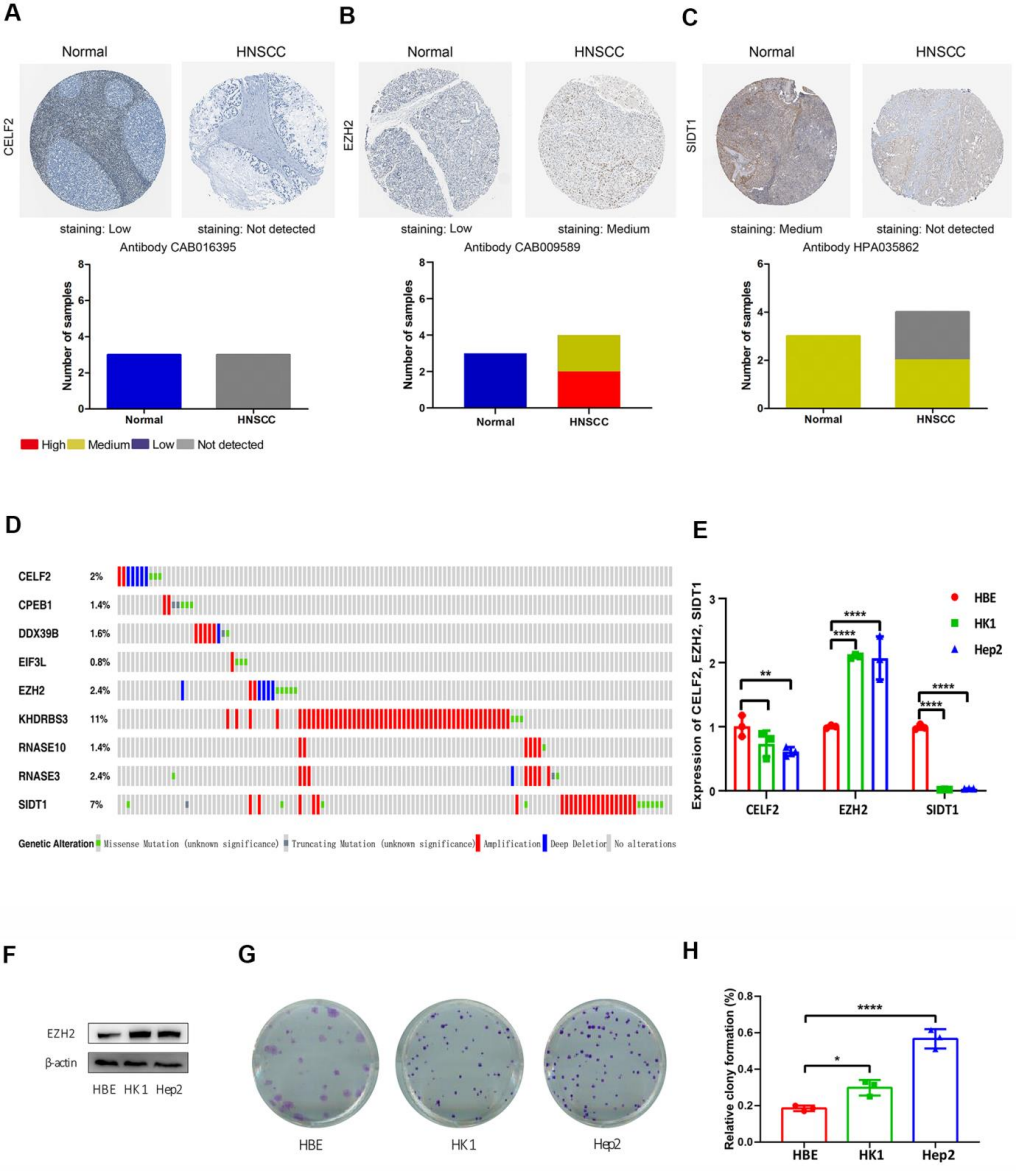
**Immunohistochemistry (IHC), mutations and verification results in the prognosis-related RBPs.** The protein levels of (**A**) CELF2, (**B**) EZH2 and (**C**) SIDT1 were determined by immunohistochemistry using indicated antibodies in HPA database, the staining strengths were annotated as Not detected, Low, Medium and High. The bar plots indicating the number of samples with different staining strength in HPA database. (**D**) OncoPrint showing the copy number alterations and mRNA expression alterations of 9 RBPs in prognostic model. (**E**) The mRNA expression levels of CELF2, EZH2 and SIDT1 in HNSCC cell lines were determined by qRT-PCR, GAPDH was verified as a housekeeping gene. (**F**) Western blot was conducted to evaluate the protein expression of EZH2 in HNSCC cell. (**G**, **H**) Colony-forming assay was conducted to evaluate the growth of HNSCC cell lines.

From the prognosis model, we acquired 9 genes and then further assessed the prognosis value of these genes in other databases. Correlation analysis of picked genes in TIMER (Tumor Immune Estimation Resource) database showed that most genes were closely associated with mRNA expression (Supplementary Table 2 and Supplementary Figure 1). The genes were analyzed by the GEPIA (Gene Expression Profiling Interactive Analysis) database using Kaplan-Meier analysis. The results show that in HNSCC, *CELF2, EZH2, RNASE10* and *SIDT1* are positively correlated with OS, indicating that these highly expressed genes correlate with a good prognosis (Figure 8). All in all, the Kaplan-Meier analysis results agree with the univariate Cox analysis results, which means that most genes are infused into specific prognosis models and have a potent predictive capacity. Next, the protein expression pattern of genes in the prognosis model were analyzed by the HPA database (Figures 9, 10A–10C). The results demonstrated that the expression of the *CELF2* protein was low in normal head and neck tissues, but this protein was not detected in HNSCC tissues (Figure 10A). The *EZH2* protein was low expressed in normal tissues and moderately expressed in tumor tissues (Figure 10B). The *SIDT1* protein was moderately expressed in normal head and neck tissues but was not detected in HNSCC tissues (Figure 10C). We then used the cBioPortal database to find out the CNV (Copy number variations) and mRNA expression changes in these genes (Figure 10D). The results demonstrated that CNVs were associated with the mRNA expression changes in these genes. It is noteworthy that SIDT1 and KHDRBS3 demonstrated the highest mRNA expression changes and CNV in the entire analysis sample, which may suggest that CNVs are the leading driving forces for the mRNA expression changes in these genes. To verify our analysis, the relative mRNA expression of *CELF2*, *EZH2* and *SIDT1* in HNSCC cell lines was evaluated by qRT-PCR. As shown in Figure 10E, compared with that in the human bronchial epithelial cell line HBE, the mRNA expression of the *CELF2 gene* was relatively lower in the laryngeal squamous carcinoma cell line Hep2 (P<0.05) but showed no difference in that in the nasopharyngeal carcinoma cell line HK1 (P>0.05). The *EZH2* mRNA expression was higher in both Hep2 and HK1 cells (P<0.01), and the *SIDT1* mRNA expression was lower in both Hep2 and HK1 cells (P<0.01); these results are consistent with the protein expression patterns detected in HNSCC tissues in HPA database. Besides, EZH2 protein expression was higher in HK1 and Hep2 than HBE cells which was verified by western blot assay as showed in Figure 10F. In addition, the clone forming ability of 2 tumor cell lines was significantly higher than that of the control cell lines (Figure 10G, 10H). Our results agreed with findings we have observed previously, which further verified the reliability of our experiment. The GSEA method was used to calculate the pathways and enriched features between low-risk and high-risk patients, as we found out that low-risk and high-risk patients have significant prognostic differences in OS. In the results of GSEA enrichment, we observed that the high-risk groups were enriched in response of EIF2AK4 GCN2 to amino acid deficiency, ribosome, SRP-dependent cotranslational protein targeting to membrane, rRNA modification in the nucleus and cytosol, Myc targets v1 and allograft rejection. (Supplementary Figure 2A–2F). The low-risk group was enriched in the transcriptional regulation by RUNX1, interferon signaling, signaling by Wnt, MAPK family signaling cascades, hallmark interferon gamma response and hallmark interferon alpha response (Supplementary Figure 2G–2L). Some studies have shown that these pathways are related to the development of HNSCC. In short, the GSEA results indicate that RBP-related signals are associated with the progression and development of HNSCC.

## DISCUSSION

Head and neck squamous cell carcinoma is a very harmful disease. The 5-year survival rate of patients is low, only 50%; in addition, the disease also affects the patients' voice, hearing, vision, etc., resulting in poor quality of life and a great burden on patients and society [15]. In recent years, lots of diagnostic molecular markers that were related to HNSCC have been discovered, but it is hard to reach accurate early detection. This may be the most significant reason of high mortality in patients. Therefore, it is pressing to develop an effectual early detection and diagnosis method to improve the treatment of HNSCC. A large number of research have reported that RBPs are dysregulated in diverse human cancers. However, little is known about the function and expression pattern of RBPs in HNSCC. Here, we extracted corresponding clinical data and sequence data from the TCGA database, used a bioinformatics analysis method to determine RBPs that was related to prognosis, and constructed a model for the prognosis of HNSCC, which may be helpful to develop biomarkers for the diagnosis and prognosis of HNSCC.

To our knowledge, this is the first time that an entire set of RBPs has been combined with HNSCC to explore and verify the potential value of RBPs in HNSCC. In our study, we searched for the expression of RBPs in HNSCC database to discover molecular biomarkers that associated with the diagnosis, treatment, and prognosis of HNSCC patients. We first screened RBPs that are differentially expressed between HNSCC and nontumor tissues, established a protein-protein interaction network of these RBPs, and obtained three key modules. Considering that these genes may be closely connected with the occurrence of HNSCC, we performed GO and KEGG analyses of these genes. Analysis of the PPI network modules showed that RBPs with different expression levels were highly enriched in the process of RNA catabolism, DNA methylation or demethylation, regulation of RNA splicing, regulation of RNA transport and mRNA monitoring. RNA plays an important role in the evolution of organisms and plays an important role in the translation of genetic information, gene expression and cell function. Studies have shown that the abnormal regulation of RNA transport, processing, translation and catabolism processes is related to the occurrence and development of various diseases [16, 17]. Epigenetics refers to changes in the expression of genetic genes unrelated to changes in the DNA sequence. DNA methylation is the most common epigenetic change. Abnormal DNA methylation can result in the activation of proto-oncogenes and the inactivation of tumor suppressor genes. The occurrence of tumors is the result of many factors, among which the activation of proto-oncogenes and the inactivation of tumor suppressor genes play vital roles. Studies have found that in many studies of head and neck tumors, methylation is often found in genes such as p16, MGM T, and DAP-kinase, and is related to the inactivation of gene expression [18, 19]. Methylation may affect the occurrence and development of head and neck tumors through the regulation of multiple genes. Splicing factors are RNA-binding proteins that affect exon selection and splicing site selection by recognizing cis regulatory elements in pre-mRNA [20]. Changes in the expression of SF may lead to an overall change in certain cancer-specific alternative splicing events, thereby affecting the occurrence and development of cancer. For example, the splicing factor SAM68 can promote the expression of BCL-XS and induce the apoptosis of leukemia cells [21]. These results indicate that RBPs affect the growth of tumor cells by regulating various biological processes, such as RNA transport, DNA methylation or demethylation, and RNA splicing.

We then constructed the model by univariate Cox regression and LASSO regression analyses and ultimately identified 9 OS-related risk genes (CELF2, CPEB1, DDX39B, EIF3L, EZH2, KHDRBS3, RNASE10, RNASE3 and SIDT1). We further created a specific prognosis model (OS model) and demonstrated that this model can provide accurate predictions for the prognosis of HNSCC patients in the training and the testing group. Besides, multivariate Cox regression analysis of the prognosis model and other clinical parameters demonstrated that the model can predict patients' prognosis independently. Moreover, GSEA of this model found that ribosomes and rRNA modifications in the nucleus and cytosol and Myc and other related signaling pathways were excessively activated in high-risk patients. It has been shown that ribosomal proteins are abnormally expressed in various tumors and affect aging, growth, apoptosis, invasion, drug resistance and radiotherapy resistance of tumor cells through various mechanisms. The proliferation of tumor cells is positively correlated with protein synthesis [22]. Some tumor suppressors indirectly regulate cell proliferation by interfering with ribosome synthesis. The inactivation of ribosomal proteins or the p53 gene promotes the synthesis of ribosomal protein in tumors and leads to cell proliferation [23]. The mutation of RPS20 affects the maturity of 18S rRNA by affecting various rRNAs and hinders the synthesis of ribosomes [24]. Carcinogenic factors can promote cell proliferation by promoting the synthesis of ribosomal proteins. In the process of tumor progression, tumor suppressors and carcinogens jointly regulate the synthesis of ribosomal proteins, which determines the direction of cell development. The nuclear oncogene Myc gene family mainly encodes proteins, and their activation and mutation can lead to cell carcinogenesis. At present, research on Myc oncogenes shows that the abnormal expression of the Myc oncoprotein is closely related to the occurrence and development of head and neck tumors [25, 26]. Low-risk group enrichment analysis showed that the enrichment of classic tumor signaling pathways such as the transcriptional regulation of RUNX1, interferon signal, Wnt and MAPK [27, 28]. RUNX1 is a transcription factor that can directly or indirectly regulate signal transduction pathways, such as the TGF-β signaling pathway, Wnt signaling pathway, and bone morphogenetic protein (BMP) signaling pathway [29–31]. With an increasing number of studies in different fields, RUNX1 plays different roles in various solid tumors. RUNX1 plays an anticancer role in esophageal cancer and gastric cancer but plays a role in promoting cancer in non-small cell lung cancer, endometrial cancer, and oral and head neck squamous cell carcinoma and plays a different role, by either inhibiting or promoting cancer, in different types of breast cancer [32].

In summary, on the basis of comprehensive analysis of corresponding clinical features and RBP expression profiles, a specific prognosis model of RBPs was determined. The genes in these models provide new targets for the treatment and intervention of HNSCC. The main limitation of this study is that the data applied in our research were acquired from several public databases. These findings need to be verified in future clinical trials. Besides, due to the limitations of the existing IHC images in the HPA (Human Protein Atlas) database, it is difficult to use statistical methods to detect the differential expression of these proteins. Future work will use local clinical specimens to detect the protein expression of selected proteins. The mechanism by which RBPs regulate the occurrence and development of HNSCC needs to be studied further. In conclusion, our research shows that differentially expressed RBPs have good diagnostic and prognostic value as biomarkers and therapeutic targets for HNSCC. Further investigations are needed to confirm our findings. Validating these models in the local clinical cohort is also important to improve the accuracy of these predictions.

## MATERIALS AND METHODS

### Data preprocessing and screening of differentially expressed RBPs

RNA sequencing data and corresponding clinical data of 502 HNSCC and 44 normal head and neck tissue specimens were acquired from the TCGA database (https://portal.gdc.cancer.gov/) (Supplementary Table 3). A total of 1542 RNA-binding proteins that have been confirmed to exist in the human genome were extracted from the literature. See Supplementary Table 4 for details. To ensure a unified standard, the RNA sequencing results (FPKM) of HNSCC were transformed and standardized by the formula log2 (x+1). The Wilcox test in R (version 4.0.0, https://www.r-project.org/) was used to calculate the differential expression of RBPs between HNSCC and control samples. The genes with at least 1.5-fold alteration and corresponding P value less than 0.05 were chosen as the differentially expressed RBPs (DERBPs).

### Gene enrichment analysis and PPI network construction and module selection

The main biological characteristics of these RBPs were detected by GO enrichment and KEGG pathway analysis. Go analysis terms include biological process (BP), molecular function (MF) and cell composition (CC). Then differently expressed RBPs are submitted to the STRING database (http://www.STRING-db.org/) to identify protein-protein interaction information. The PPI network is further constructed and visualized by using the software of Cytoscape 3.6.1. The important modules and genes were screened out in PPI network by using molecular complex detection (mcode) plug-in. The MCODE scores and the number of nodes are both greater than 5. Statistically significant differences P<0.05 are denoted as starred values.

### Construction and verification of prognosis model in HNSCC

We first integrated the expression data of DERBPs with the corresponding clinical information, selected RBPs co-expressed in the PPI network, and then randomly divided the data into a testing group and a training group for following verification. Univariate Cox regression analysis was used to analyze the expression data of RBPs in the training group, and RBPs that were significantly related to survival were obtained (P <0.05). Finally, we used lasso regression and multivariate Cox regression analysis to constructed a prognosis model. In the training group and the testing group, the risk score of each patient was calculated according to the regression coefficient and expression value of each gene in the model. The calculation formula is: β1 * Exp1 + β2 * exp2 + βi * EXPi, in which β represents coefficient value and exp represents gene expression level. The risk score is an indicator to measure the prognostic risk of each HNSCC patient. We used the median risk score to draw a Kaplan-Meier survival curve and generated receiver operating characteristic (ROC) curves to determine the accuracy of the prediction model. Finally, the rms R package was used to analyze the nomogram plot and predict the possibility of OS. P <0.05 is a significant difference.

### Comprehensive analysis of RBPs in HNSCC prognosis model

Correlation analysis was performed on the selected RBPs in the TIMER database, and the Pearson correlation coefficient between each gene pair was calculated. Then use the GEPIA2 database (http://gepia.cancer-pku.cn/) to perform Kaplen-Meier analysis on the RBPs in the risk-specific model, and use the log-rank test to determine the statistical significance. The expression of RBPs at translation level was analyzed by comparing the immunohistochemical staging images in HPA database (http://www.protein.atlas.org/). On the basis of the staining intensity, it is marked as high, medium, low and undetected. The cbioProtal database (http://www.cbioportal.org/) was used to further analyze the nine RBPs in the risk-specific model to evaluate alterations in mRNA expression and copy number. Last, GSEA (gene set enrichment analysis) was executed to examine the ways and characteristics of enrichment in the predicted low-risk and high-risk population. Using GSEA, this research studied whether the characteristics of activation/inhibition genes were abundant in low-risk and high-risk patients. The standardized enrichment score (NES) and standardized P value are used to calculate the enrichment of the Hallmarks and canonical pathways. Terms with | NES |> 1 and P <0.05 are considered significantly rich.

### Verification of screened RBPs by qRT-PCR

The human bronchial epithelial cell line HBE, laryngeal squamous carcinoma cell Hep2 and nasopharyngeal carcinoma cell line HK1 were obtained from the Shanghai Zhong Qiao Xin Zhou Biotechnology. Hep2 and HK1 cells were maintained at 37° C in a 5% CO_2_ incubator in Dulbecco’s modified Eagle’s medium (DMEM) with 10% fetal bovine serum (FBS) (Biological Industries, Kibbutz Beit Haemek, Israel) and 1% penicillin–streptomycin (Keygen Biotech, Nanjing, China). HBE cells were cultured with specific keratinocyte medium supplemented with 1% keratinocyte growth factor and 1% penicillin–streptomycin (Zhong Qiao Xin Zhou Biotech, Shanghai, China). For quantitative analyses of screened RBPs, total RNA was extracted using TrizoL (Life Technologies, Carlsbad), and cDNA was prepared using High Capacity cDNA Reverse Transcription Kit (Thermo Fisher, MA) and qPCR were performed with NovoStart® SYBR qPCR SuperMix Plus (Novoprotein, Shanghai, China). The qPCR primer sequences of EZH2 gene were 5’-CCCGCTGAGGATGTGGATAC-3’ and 5’-CATGGTTAGAGGAGCCGTCC-3’, primer sequences of SIDT1 were 5’-AGCCCCTCTCAACCTCAGTA-3’ and 5’-GCAGCTTTCTTGGTCATGGA-3’, primer sequences of CELF2 were 5’-CAGCACCAATGCAAACCCTC and TCCCGAGAGAGGTCAAGGAG-3’, which were designed by Primer Premier 5.

The quality of cDNA samples was verified using GAPDH as a housekeeping gene.

### Verification of screened RBPs by western blot

Protein lysates were prepared using RIPA lysis buffer (0.1% SDS, 1% NP-40, 1 mM EDTA, 50 mM Tris PH 7.5, 150 mM NaCl, 0.25% deoxycholate) with protease and phosphatase inhibitors (Roche, Welwyn Garden City, UK). Protein concentration was determined using BCA Protein Assay Kit (Beyotime, Shanghai, China). Following 10% SDS gel electrophoresis and subsequent immunoblotting, bound anti-EZH2 antibody (1:1000), protein expression was detected by ECL Chemiluminescence substrate (Biosharp, Shanghai, China).

### Verification of screened RBPs by colony-forming assay

HBE, HK1 and Hep2 cells were diluted and plated into six-well plates in triplicate to execute a colony-formation assay (CFA). Inoculate 200 cells into each well of a 6-well plate, change the medium every 3 days, and culture for 2 weeks. After that, the colonies containing more than 100 cells were fixed by methanol, stained using 0.3% crystal violet (Sigma, USA) and counted manually. And the colony formation rate was reckoned using formula as below: colony formation rate = (number of colonies / number of seeded cells) × 100%.

## Supplementary Material

Supplementary Figures

Supplementary Table 1

Supplementary Table 2

Supplementary Table 3

Supplementary Table 4
