# Supplementary material for: Development and validation of a RNA binding protein-associated prognostic model for head and neck squamous cell carcinoma
Source: Aging (Albany NY). 2021 Mar 24;13(6):7975–97. doi: 10.18632/aging.202848 (PMC8034976; doi:10.18632/aging.202848)
Supplement: Supplementary Figures [file aging-13-202848-s001.pdf]

SUPPLEMENTARY FIGURES

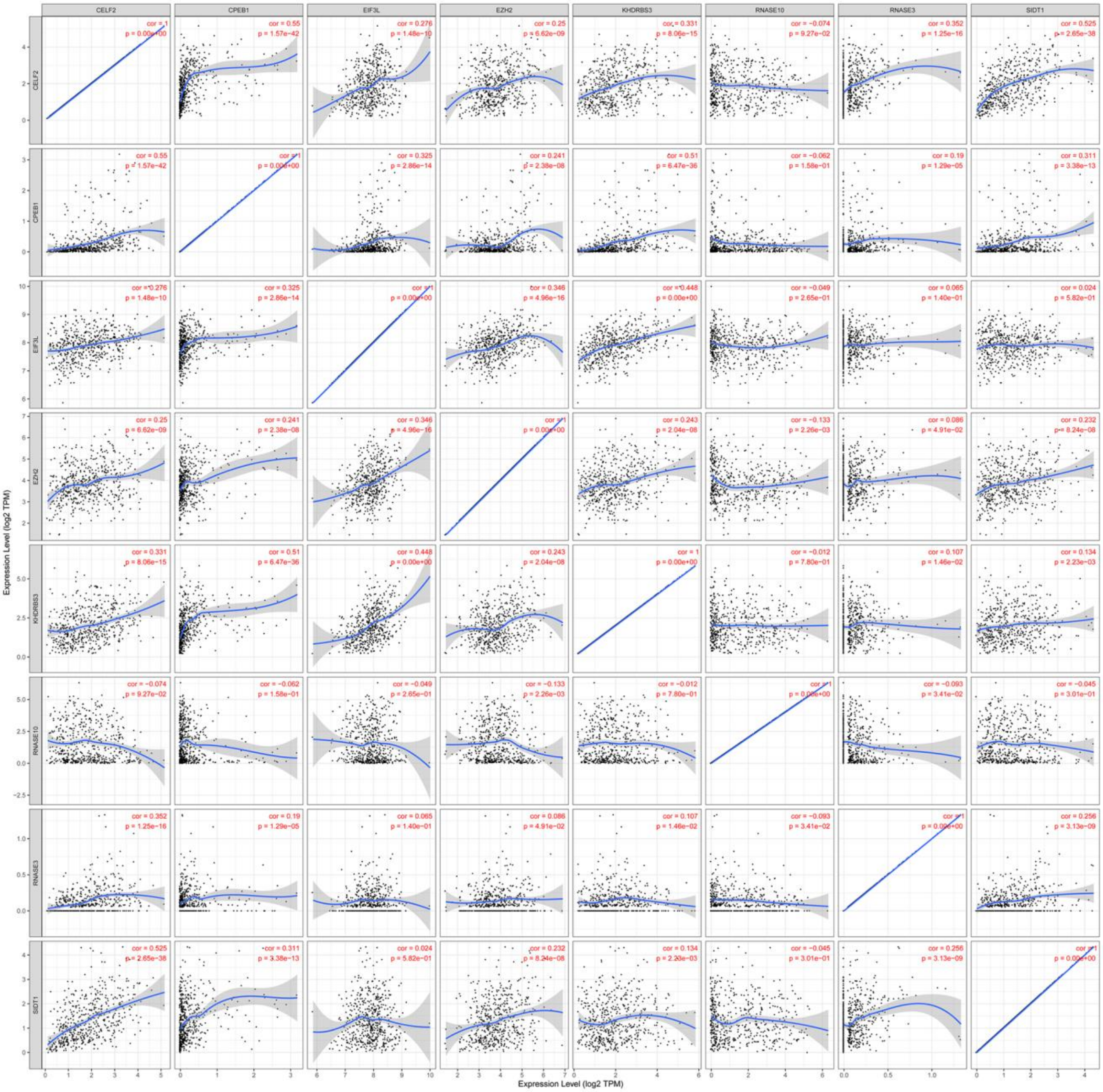

Supplementary Figure 1. The relationship between the expression of RBPs in prognostic model. Cor: Correlation coefficient. The value range of correlation coefficient is (- 1, 0) or (0, 1). When the value range is (- 1, 0), it means negative correlation, when the value range is (0, 1), it means positive correlation.

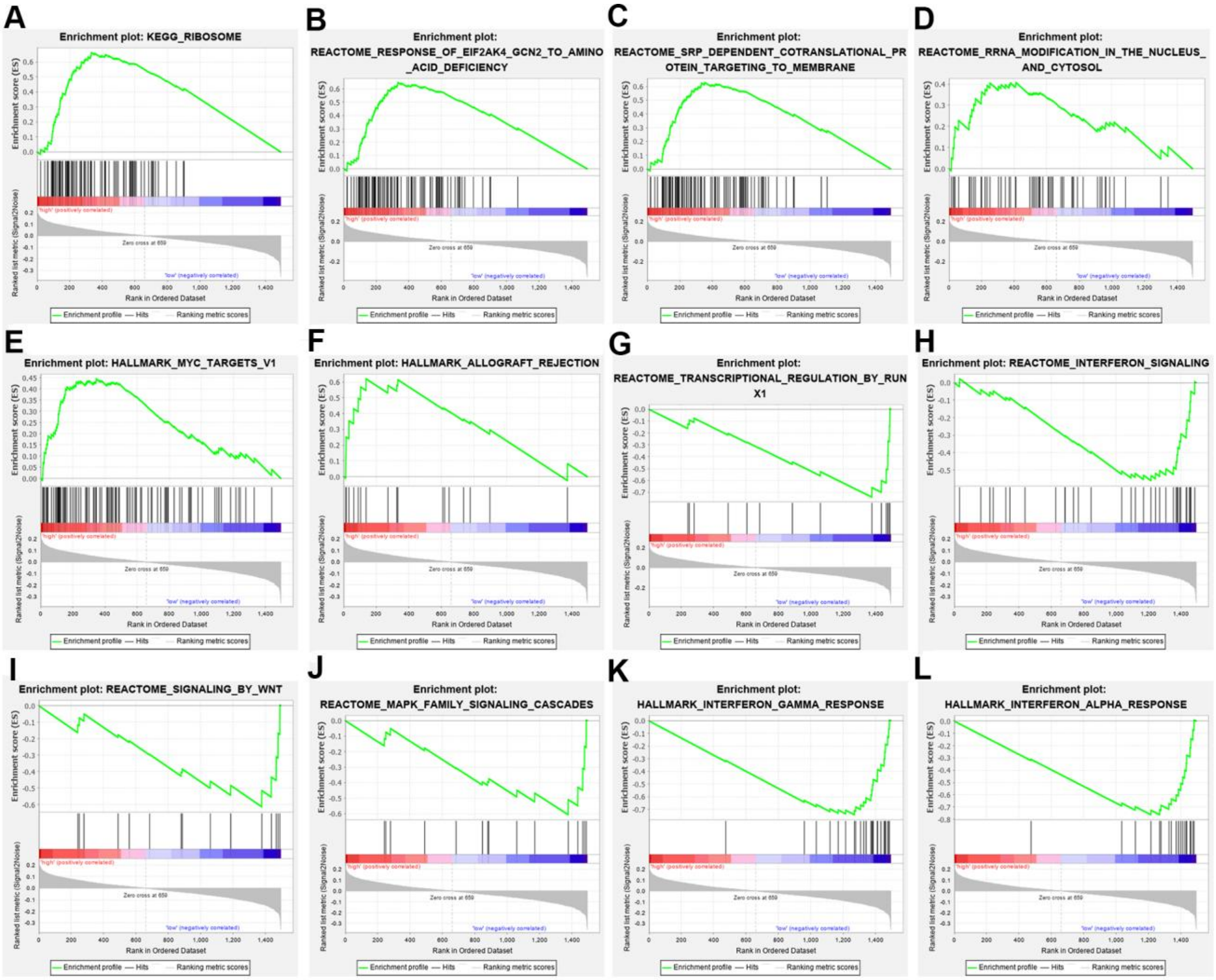

Supplementary Figure 2. Gene set enrichment analysis (GSEA) of curated genesets and hallmark genesets in high-risk and low-risk patients with HNSCC. (**A**–**F**) GSEA results in high-risk group, (**G**–**L**) GSEA results in low-risk group.

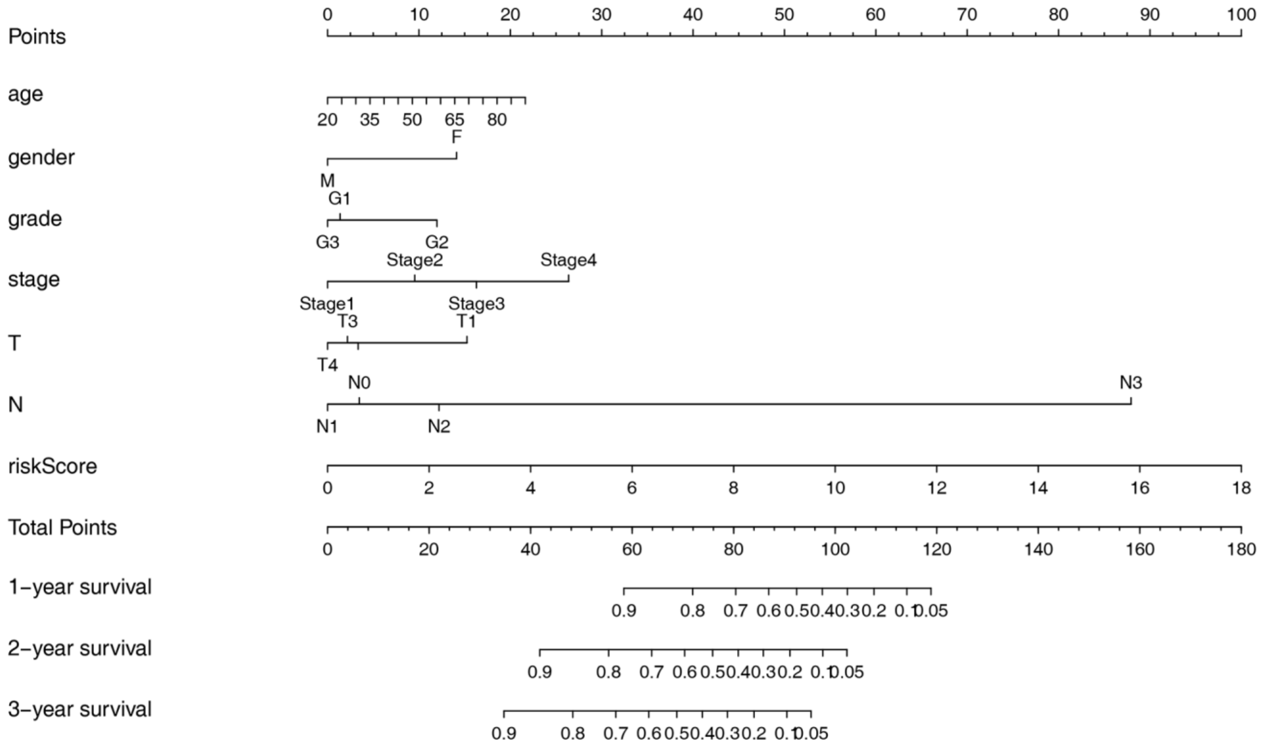

Supplementary Figure 3. Nomogram for predicting 1-, 3-, and 5-year OS of HNSCC patients.
