# Supplementary material for: Development and validation of a RNA binding protein-associated prognostic model for head and neck squamous cell carcinoma
Source: Aging (Albany NY). 2021 Mar 24;13(6):7975–97. doi: 10.18632/aging.202848 (PMC8034976; doi:10.18632/aging.202848)
Supplement: Supplementary Table 2 [file aging-13-202848-s003.pdf]

Supplementary Table 2. Correlation analysis of RBPs in prognosis model.

var X	var Y	Cor	P value
CELF2	CELF2	1	0
CELF2	CPEB1	0.549586	1.57E-42
CELF2	SIDT1	0.524933	2.65E-38
CELF2	RNASE3	0.351555	1.25E-16
CELF2	KHDRBS3	0.331097	8.06E-15
CELF2	EIF3L	0.275659	1.48E-10
CELF2	EZH2	0.250421	6.62E-09
CELF2	RNASE10	-0.07367	0.092696
CPEB1	CPEB1	1	0
CPEB1	CELF2	0.549586	1.57E-42
CPEB1	KHDRBS3	0.510051	6.47E-36
CPEB1	EIF3L	0.324573	2.86E-14
CPEB1	SIDT1	0.311356	3.38E-13
CPEB1	EZH2	0.241256	2.38E-08
CPEB1	RNASE3	0.189666	1.29E-05
CPEB1	RNASE10	-0.06186	0.158187
EIF3L	EIF3L	1	0
EIF3L	KHDRBS3	0.448004	0
EIF3L	EZH2	0.345825	4.96E-16
EIF3L	CPEB1	0.324573	2.86E-14
EIF3L	CELF2	0.275659	1.48E-10
EIF3L	RNASE3	0.064712	0.139812
EIF3L	RNASE10	-0.04887	0.265031
EIF3L	SIDT1	0.024124	0.582364
EZH2	EZH2	1	0
EZH2	EIF3L	0.345825	4.96E-16
EZH2	CELF2	0.250421	6.62E-09
EZH2	KHDRBS3	0.243148	2.04E-08
EZH2	CPEB1	0.241256	2.38E-08
EZH2	SIDT1	0.232025	8.24E-08
EZH2	RNASE10	-0.13339	0.002258
EZH2	RNASE3	0.086188	0.049055
KHDRBS3	EIF3L	0.448004	0
KHDRBS3	KHDRBS3	1	0
KHDRBS3	CPEB1	0.510051	6.47E-36
KHDRBS3	CELF2	0.331097	8.06E-15
KHDRBS3	EZH2	0.243148	2.04E-08
KHDRBS3	SIDT1	0.133576	0.002226
KHDRBS3	RNASE3	0.106858	0.014583
KHDRBS3	RNASE10	-0.01227	0.77979
RNASE10	RNASE10	1	0
RNASE10	EZH2	-0.13339	0.002258
RNASE10	RNASE3	-0.09274	0.034139
RNASE10	CELF2	-0.07367	0.092696
RNASE10	CPEB1	-0.06186	0.158187
RNASE10	EIF3L	-0.04887	0.265031
RNASE10	SIDT1	-0.04534	0.301168

RNASE10	KHDRBS3	-0.01227	0.77979
RNASE3	RNASE3	1	0
RNASE3	CELF2	0.351555	1.25E-16
RNASE3	SIDT1	0.255608	3.13E-09
RNASE3	CPEB1	0.189666	1.29E-05
RNASE3	KHDRBS3	0.106858	0.014583
RNASE3	RNASE10	-0.09274	0.034139
RNASE3	EZH2	0.086188	0.049055
RNASE3	EIF3L	0.064712	0.139812
SIDT1	SIDT1	1	0
SIDT1	CELF2	0.524933	2.65E-38
SIDT1	CPEB1	0.311356	3.38E-13
SIDT1	RNASE3	0.255608	3.13E-09
SIDT1	EZH2	0.232025	8.24E-08
SIDT1	KHDRBS3	0.133576	0.002226
SIDT1	RNASE10	-0.04534	0.301168
SIDT1	EIF3L	0.024124	0.582364
